# Downregulation of Autophagy-Related Proteins 1, 5, and 16 in Testicular Germ Cell Tumors Parallels Lowered LC3B and Elevated p62 Levels, Suggesting Reduced Basal Autophagy

**DOI:** 10.3389/fonc.2018.00366

**Published:** 2018-09-07

**Authors:** He Liu, Zhaoyue He, Peter Bode, Holger Moch, Hans-Uwe Simon

**Affiliations:** ^1^Institute of Pharmacology, University of Bern, Bern, Switzerland; ^2^Department of Pathology and Molecular Pathology, University and University Hospital Zurich, Zurich, Switzerland

**Keywords:** testicular germ cell tumor (TGCT), ATGs, LC3B, p62, autophagy, tumor suppression

## Abstract

Autophagy is a cellular “self-digestion” process known to be essential for various physiological and pathological pathways, including cancer, where its role appears to be context-dependent. In this work, we aimed to investigate the level of autophagy by evaluating the expression of key autophagy-related proteins (ATGs) in testicular germ cell tumors (TGCT) for which autophagy has been rarely investigated. We decided to use an immunohistochemical (IHC) staining approach employing a tissue microarray (TMA). Software-based evaluation of the integrated optical densities (IODs) of these proteins indicated a significant downregulation of ATG1, ATG5, and ATG16L1. Accordingly, reduced levels of microtubule-associated proteins 1A/1B light chain 3B (LC3B) were found to parallel increases in sequestosome-1 (SQSTM1 or p62), a protein normally degraded via autophagy, suggesting an *in vivo* reduction in autophagy with TGCT. Thus, our work provides evidence for a tumor suppressive function of autophagy in the development of TGCT and supports the concept of a context-dependent role of autophagy in tumorigenesis which is tumor type-dependent.

## Introduction

Autophagy as a cellular catabolic process is essential at a basal level to ensure normal cellular homeostasis by the controlled degradation of cellular contents (1, 2). It is highly regulated by the so-called ATGs as well as by class III PI3K and mTOR pathways (3). Under most stress conditions, for example a shortage of nutrients, autophagy is induced to cope with starvation by degrading cellular proteins. Induction of autophagy is often observed in cancer cells experiencing an anti-cancer therapy which involves drastic metabolic disturbances and/or DNA damage (4). Therefore, inhibition of autophagy has been proposed as a combination with conventional anti-cancer therapies for improving efficacy of the anti-cancer treatment and/or to overcome drug resistance (3, 4). However, it is important to note the potential for dual roles of autophagy in cancer, both as “friend” and “foe” (3–5).

Our previous work showed a downregulation of ATG5 and autophagy in melanoma and provided evidence that enforced downregulation of ATG5 accelerates cell proliferation by delaying oncogene-induced senescence (6–8). A protective function of autophagy in early tumorigenesis has been demonstrated in an experimental tumor model (9). Interestingly, a dual role for autophagy was also shown in a mouse lung tumor model, autophagy-deficient mice showing accelerated tumor initiation, but prolonged overall survival (10).

Given the importance of autophagy in tumorigenesis and anti-cancer therapy, we decided to investigate the level of autophagy in TGCT for which the role of autophagy has been little documented. IHC staining of the key autophagy-regulating proteins followed by software-based quantification of the integrated optical densities (IODs) of the corresponding images revealed a reduction in ATG1, ATG5, and ATG16L1 expression. Accordingly, we found also a reduced level of LC3B accompanied by an increased level of p62, indicating diminished basal autophagy in TGCT.

## Materials and methods

### IHC staining

The TMA was constructed by the Department of Pathology and Molecular Pathology, University and University Hospital Zurich. The study was approved by the Cantonal Ethics Committee of Zurich (KEK-ZH-Nr. 2014-0604). The retrospective use of tumor tissues of patients is in accordance with the Swiss Law (“Humanforschungsgesetz”), which, according to Article 34, allows the use of biomaterial and patient data for research purposes without informed consent under certain conditions that include the present cases. Law abidance of this study was reviewed and approved by the Ethics Commission of the Canton Zurich. IHC was performed as previously described (6). The following antibodies were used: anti-ATG1 (AP8104b, Abgent), anti-ATG5 (11C3, Nanotools), anti-ATG16L1 (LS-B2723, Lifespan Biosciences), anti-LC3B (023-100, Nanotools), and anti-p62 (P0067, Sigma).

### Quantification of the staining intensity

The staining intensities of these proteins were quantified as IODs using Image Pro Plus software as previously described (6).

### Statistics

Statistical analysis was performed using the unpaired Student *t*-test or ANOVA followed by the Bonferroni test for multiple comparisons as indicated.

## Results

### Reduced expression of ATGs in TGCT

**Figure 1:**
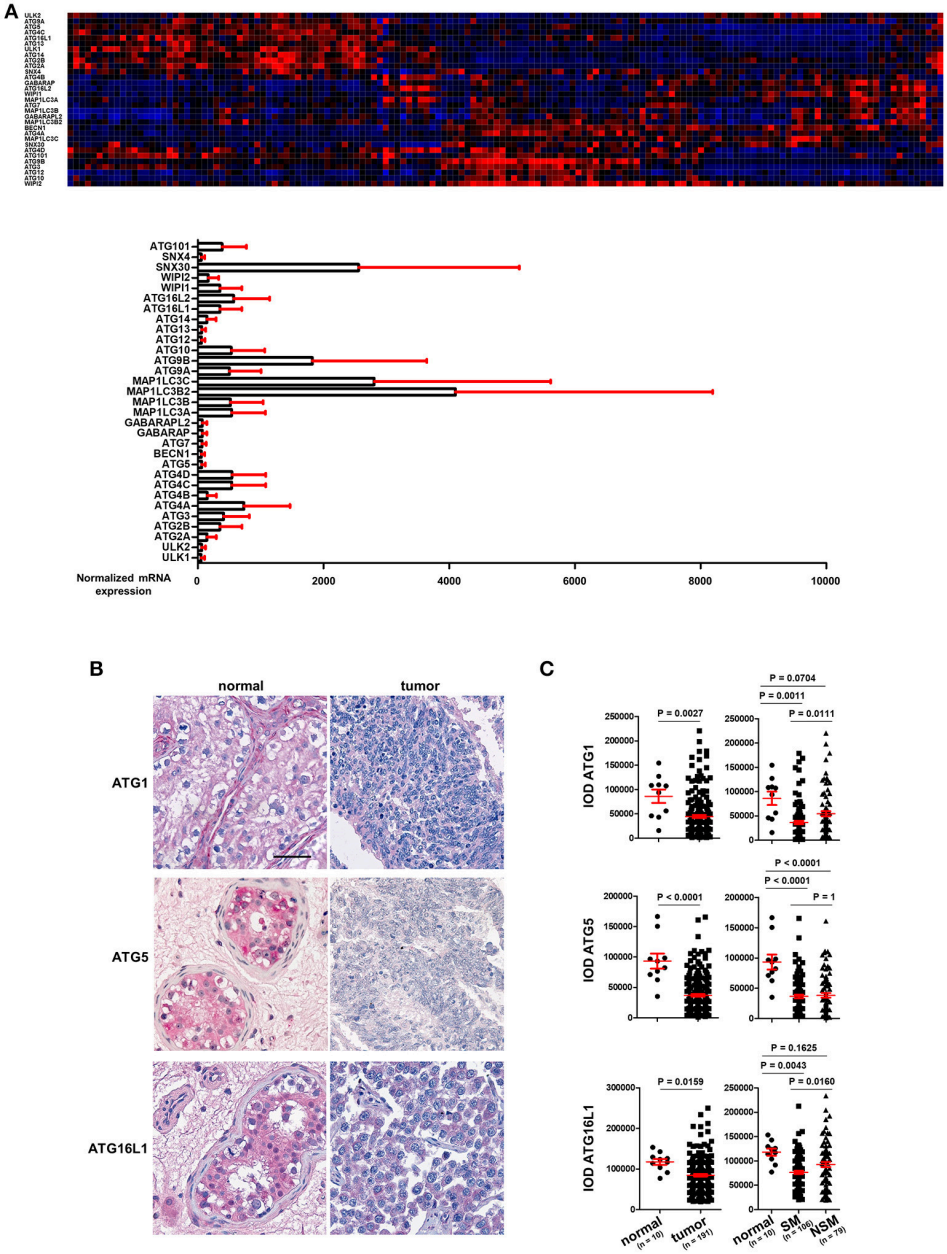
ATG1, ATG5, and ATG16L1 are downregulated in TGCT. **(A)** The mRNA expression of ATGs in TGCT. mRNA expression data of ATGs in TGCT (*n* = 157) were extracted from the TCGA consortium data base and are presented as a heat map as well as bar diagrams. **(B)** Representative images of IHC of ATG1, ATG5, and ATG16L1. Scale bars: 50 μm. **(C)** Quantification of the IODs of these proteins using Image Pro Plus software. ANOVA followed by the Bonferroni test was performed with data shown on the right panel.

TGCT are derived from primordial germ cells/gonocytes that failed to differentiate into spermatogonia. They are classified as seminomas or non-seminomas and display a wide range of histological subtypes. Seminomas possess pluripotent potential, but are more indolent, whereas non-seminomas are often differentiated and more aggressive (11–13). mRNA expression data for the *ATGs* in TGCT was extracted from the TCGA consortium data base (https://cancergenome.nih.gov/) and is presented in Figure 1A (*n* = 157). The individual *ATGs* show a differential expression in these cancer tissues (Figure 1A). However, whether any *ATG* mRNA level differed between cancer and normal tissues could not be concluded owing to a lack of normal testis tissue samples.

With TMAs containing punches of 10 non-neoplastic testicular tissues retrieved from contralateral testicular biopsies of patients with TGCT and punches from 191 TGCT tumor patients, we employed immunohistochemical methods to stain for key autophagy regulators, ATG1, ATG5, and ATG16L1. Tumors were classified according to the 2016 WHO classification (11). Homogenous and intense staining of these proteins was observed in the parenchyma of normal testis tissues. However, the staining intensities were consistently and strongly reduced in TGCT cells (Figure 1B). Quantification of the IODs of these proteins showed a significant reduction in the expression of ATG1, ATG5, and ATG16L1 (Figure 1C). ATG1 and ATG16L1 are even further reduced in seminoma (SM) compared to non-seminoma (NSM) (Figure 1C). In contrast, the expression of ATG5 did not differ significantly between the two subtypes (Figure 1C). Taken together, the reduced expression of ATGs in TGCT strongly suggest a possible decrease in autophagy, which might promote survival of gonocytes (14).

### Reduced level of autophagy in TGCT

**Figure 2:**
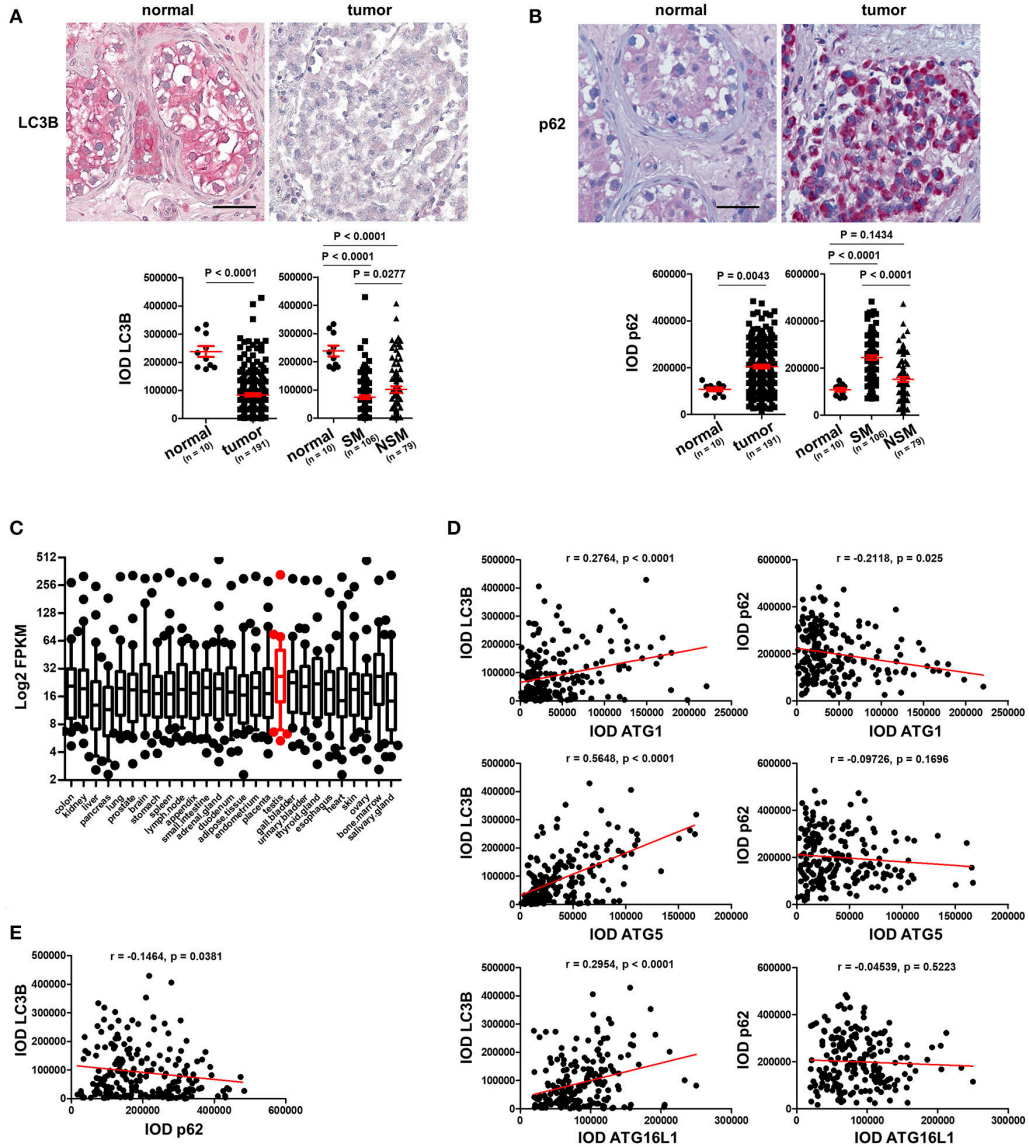
Basal level of autophagy is reduced in TGCT. **(A)** Representative images of IHC staining of LC3B and its quantification. Scale bars: 50 μm. **(B)** Representative images of IHC staining of p62 and its quantification. Scale bars: 50 μm. **(C)** Log2 FPKM of the gene expression data of 28 ATGs in 27 normal tissues based on findings in a previous publication (15). **(D)** The IODs of these proteins were applied to Prism software for correlation analysis between ATGs and LC3B or p62. The Pearson *r* values and *p*-values obtained from the software are presented. **(E)** Correlation between p62 and LC3B was analyzed and presented like described in **(D)**.

To prove our hypothesis that autophagy is reduced in TGCT, we stained the TMAs with an antibody against LC3B, a protein which is required for formation of the characteristic organelles called autophagosomes, in which engulfed cellular contents are degraded (3). Homogenous and strong staining in the parenchyma was observed in normal testicular tissues in contrast to tumor tissues that showed instead a rather weak staining of LC3B (Figure 2A). Quantification of IODs of LC3B demonstrated a significant reduction of LC3B expression in TGCT compared to normal tissues (Figure 2A). Since autophagy is a dynamic process and increased amounts of autophagosomes or upregulation of LC3B could also be the consequence of inhibited autophagosome degradation (3), we investigated the levels of p62, which is normally degraded via autophagy (3). Interestingly, consistently weak staining of p62 was observed in normal parenchyma of the testicular tissue, whereas it is strongly stained in the tumor tissue (Figure 2B). Quantification of IOD of p62 demonstrated a significant increase in p62 in tumor compared with normal tissues (Figure 2B). Moreover, based on a previous publication (15), we specifically analyzed the expression of 28 ATGs in 27 different normal tissues. These data showed that the 28 ATG genes are highly expressed (high FPKM values) in normal testis tissue, suggesting that the testis exhibits a high level of autophagy (Figure 2C). Taken together, these data indicate a reduced autophagic activity in TGCT probably owing to downregulation of the ATGs.

### The expression of ATG proteins correlates with markers of autophagic activity

To test our hypothesis that the reduced level of autophagy in TGCT possibly resulted from downregulation of key ATGs, we performed a correlation analysis between the expression levels of ATGs to those of LC3B and p62. As shown in (Figure 2D), the expression of the three investigated ATGs correlated significantly with LC3B expression, especially ATG5 (Figure 2D). However, only ATG1, but not ATG5 or ATG16L1 negatively correlated with p62 (Figure 2D). Additionally, p62 levels correlated negatively with those of LC3B (Figure 2E). Although not causal, this correlation analysis does give a strong hint that the reduced basal level of autophagy in TGCT is at least partially owing to a reduction in the expression of the investigated ATGs.

## Discussion

Analyzing autophagy in tissues remains difficult. Due to the dynamic features of autophagy it is important to consider levels of both LC3B and p62 as well as other ATGs in order to obtain a general picture of the autophagic network. The analysis of large cohorts of patients clearly helps to obtain conclusive data. It must be noted that the expression levels of LC3B and/or p62 do not necessarily reflects the level of autophagy (16). For an adequate interpretation of the level of autophagy, autophagy inducers, and/or inhibitors must be applied to monitor autophagic flux. For a retrospective study of autophagy, differences in patients' cohorts and experimental procedures, especially the source of the antibodies, may also lead to different conclusions. For example, in another study, no significant difference in autophagy was found between non-neoplastic and TGCT patients using a p62 antibody different from ours (17). Nevertheless, our evaluation of the expression of ATGs as well as LC3B and p62 strongly suggests a reduction in autophagy in TGCT and points to a possible inhibitory function of the autophagic pathway in the tumorigenesis of TGCT.

## Conclusion

We have observed reduced expression of ATG1, ATG5, and ATG16L1 as well as decreased LC3B and elevated p62 levels, suggesting a diminished basal autophagy in TGCT and a likely tumor-suppressive function of autophagy in the tumorigenesis of TGCT.

## Author contributions

HL, ZH, and HUS designed the research. HL performed IHC. ZH quantified images using bioinformatics tools and evaluated the data with HL. PB and HM constructed TMA. HL and HUS wrote the manuscript. All authors read and approved the final manuscript.

### Conflict of interest statement

The authors declare that the research was conducted in the absence of any commercial or financial relationships that could be construed as a potential conflict of interest.

## Abbreviations

ATG: Autophagy-related proteins
TGCT: testicular germ cell tumor
TMA: tissue microarray
IOD: integrated optical density
LC3B: microtubule-associated proteins 1A/1B light chain 3B
sequestosome-1: SQSTM1 or p62
PI3K: phosphoinositide 3-kinase
mTOR: mammalian target of rapamycin
IHC: immunohistochemistry
TCGA: The Cancer Genome Atlas
SM: seminoma
NSM: non-seminoma
FPKM: fragments per kilobase million.
